# Immunotherapy for Epstein-Barr Virus-Related Lymphomas

**DOI:** 10.4084/MJHID.2009.010

**Published:** 2009-11-17

**Authors:** Alana A. Kennedy-Nasser, Catherine M. Bollard, Helen E. Heslop

**Affiliations:** Center for Cell and Gene Therapy, Baylor College of Medicine, Texas Children’s Hospital and The Methodist Hospital, Houston

## Abstract

Latent EBV infection is associated with several malignancies, including EBV post-transplant lymphoproliferative disorders (LPD), Hodgkin and non-Hodgkin lymphomas, nasopharyngeal carcinoma and Burkitt lymphoma. The range of expression of latent EBV antigens varies in these tumors, which influences how susceptible the tumors are to immunotherapeutic approaches. Tumors expressing type III latency, such as in LPD, express the widest array of EBV antigens making them the most susceptible to immunotherapy. Treatment strategies for EBV-related tumors include restoring normal cellular immunity by adoptive immunotherapy with EBV-specific T cells and targeting the malignant B cells with monoclonal antibodies. We review the current immunotherapies and future studies aimed at targeting EBV antigen expression in these tumors.

## Introduction:

By adulthood, over 95% of individuals have been infected with Epstein-Barr virus (EBV), which can cause either a mild, self-limiting infection in childhood or infectious mononucleosis in adolescents. EBV enters the body via the oropharynx and infects resting B cells and/or epithelial cells^1^. Because these B cells are highly immunogenic, they induce an expansion of virus-specific and nonspecific T cells that results in regression of infected B cells; however, a small number of B cells express only a limited array of less immunogenic EBV antigens, such as EBNA-1 and in some cases express no EBV antigens, allowing these EBV-infected B cells to evade the immune response so that the virus can persist in latency for the life of the individual^2^. Reactivations can occur, but are usually readily controlled by the EBV-specific immune response.

## EBV-Related Malignancies:

**Figure 1.**
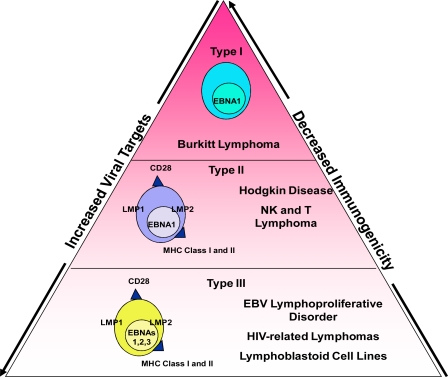
Types of EBV Latency

Latent EBV is associated with a heterogeneous group of lymphoid malignancies, including Hodgkin disease (HD), NK and T cell lymphomas, Burkitt lymphoma and lymphoproliferative disorders (LPDs) 3–5. While all are EBER positive, the EBV latent protein expression varies, and three distinct types of EBV latency have been characterized with type I being least immunogenic and type III the most immunogenic^3^ (Figure 1). Type III latency tumors include LPDs which have the same phenotype as *in vitro* generated lymphoblastoid cell lines (LCLs) and occur in immunocompromised hosts. These tumors express a full array of latent EBV antigens (EBNA-1, 2A, 2B, 3A, 3B, 3C, LP, and LMP1 and 2) and major histocompatibility complex (MHC) class I/II and costimulatory molecules, making them highly immunogenic and susceptible to immunotherapy. Type II latency (HD and NK/T lymphomas) express a more restricted EBV antigen expression pattern including the subdominant EBV antigens, LMP1 and LMP2, but also express MHC Class I/II and costimulatory molecules. These tumors generally arise in the immunocompetent host and employ multiple immune evasion strategies including restricted antigen expression. Type I latency (Burkitt lymphoma) is defined by the presence of EBNA-1 without expression of other latent antigens; thus, these tumors are the least immunogenic and therefore the least susceptible to T-cell immunotherapy.

## Immunotherapy For Type Iii Latency Tumors:

The balance between EBV-derived B-cell proliferation and cellular immunity that exists in normal hosts may be altered in immunocompromised hosts so that EBV-LPD can occur. The onset of LPD is often preceded by viral reactivation and increased numbers of latently infected B cells in peripheral blood^6^, as detected by elevated levels of EBV DNA in peripheral blood or plasma by polymerase chain reaction^7^–^9^. Monitoring of viral loads is therefore a sensitive means of monitoring patients at risk of developing LPD but the specificity varies with different clinical scenarios and many immunodeficient patients will have an increase in circulating EBV-infected B cells without developing LPD^10^,^11^.

### Post-transplant EBV-associated Lymphoproliferative Disorder:

Post transplant EBV-LPD can occur following either hematopoietic stem cell transplant (HSCT) or solid organ transplant (SOT) due to the immune suppression required to prevent graft-versus-host disease (GvHD) or rejection and the risk is related to the degree of immune supression^12^. The development of LPD is strongly associated with a defective T-cell immune response to EBV but other immunologic factors such as cytokine polymorphisms may also influence the risk^13^.

In HSCT the highest incidence of EBV-LPD is seen in the first 3 to 6 months prior to T-cell immune recovery. Whereas EBV-specific cellular immunity is rapidly re-established in unmanipulated, matched sibling graft recipients, immune reconstitution is significantly delayed in patients receiving T-cell depleted grafts, unrelated or mismatched related donor grafts or recipients who receive T-cell depleting antibodies in vivo^14^,^15^. Hence, the risk of developing EBV-LPD varies with different stem cell sources and manipulation with those receiving stem cells from unrelated or HLA-mismatched unrelated donors having the greatest risk, due to either T-cell depletion of the graft or administration of T-cell depleting antibodies to prevent GvHD. However, depletion methods using Campath-1H (anti-CD52) remove both T and B cells and is associated with lower rates of EBV^16^,^17^. EBV-LPD post HSCT is typically of donor origin, while EBV-LPD post SOT generally arises from recipient hematopoietic cells although can arise from transferred B cells in the grafted organ. The overall incidence of EBV-PTLD after SOT is less than 1% but can be as high as 31%, depending on the organ transplanted and the level of immune suppression^18^.

### CD20 Monoclonal Antibody Therapy:

Immunotherapies to prevent and treat EBV revolve around two crucial concepts: 1) removal of EBV-infected B cells or 2) expansion of EBV-specific cell-mediated immunity. The first anti-B-cell antibodies used to target EBV-infected B cells were monoclonal antibodies against CD21, the receptor used by EBV to enter B cells, and CD24, an antigen expressed by B-cells and granulocytes, and some success was reported – 57% complete remission, with 35% long-term survival (follow-up, 35–72 months).^19^,^20^ However, the effects of subsequent therapy were short lived, with the rapid re-emergence of B cells (and EBV-LPD in many cases) after treatment cessation.

Over the past 9 years the CD20 humanized antibody (rituximab) has been increasingly used in the EBV-LPD setting^21^. Since CD20 is a cell surface antigen present on all circulating B cells, this long-acting antibody may result in B cell depletion that persists for over six months. Many centers use this antibody as prevention or treatment of EBV-LPD post HSCT with response rates varying between 55% and 100%^15^,^22^–^24^. However, relapse can still occur after B cell recovery since rituximab does not restore cellular immunity to EBV^9^.

### Donor Lymphocyte Infusions:

The simplest T-cell immunotherapeutic approach to treat viral infections post HSCT is the use of unmanipulated donor lymphocyte infusions (DLI), which can be easily obtained via a simple blood draw. Since most EBV-seropositive individuals have a high frequency of EBV-specific precursors, the transfer of unmanipulated DLI should restore the immune response to EBV. While DLI infusions post HSCT can effectively eradicate EBV-LPD as early as 2 to 4 weeks post infusion^25^, the risk of graft-versus-host disease (GvHD) due to alloreactivity makes DLI treatment for EBV-LPD a less attractive option than more specific EBV therapies.

### Donor-derived EBV-CTL:

To avoid the risk of alloreactivity observed with DLI, donor-derived EBV-specific CTL can be generated in the laboratory for adoptive immunotherapy. Since EBV-CTL circulate in normal donors, *ex vivo* expansion of the EBV-CTL is feasible for patients post HSCT. Polyclonal EBV-CTL lines for clinical use can be selectively generated in the laboratory by stimulating donor peripheral blood mononuclear cells (PBMC) with donor-derived, EBV-transformed B lymphoblastoid cells lines, which act as highly effective antigen presenting cells^26^–^29^. These EBV-CTLs contain both CD4- and CD8-positive T cells that recognize multiple latent and lytic viral antigens. EBV-CTL infusions to prevent or eradicate EBV infection have been very efficacious in the post HSCT setting. We have recently reviewed the long term follow up on 114 patients who had received infusions of EBV-specific cytotoxic T lymphocytes (CTLs) at three different centers to prevent or treat EBV-positive lymphoproliferative disease (LPD) arising after hematopoietic stem cell transplantation^30^. Of the 101 patients who received CTL prophylaxis, none developed EBV-positive LPD^30^. 13 patients were treated with CTLs for biopsy-proven or probable LPD and 11 achieved sustained complete remissions^30^. Several other groups have also confirmed the activity of EBV-CTLs in treating LPD following transplant including LPDs persisting after treatment with Rituximab^31^,^32^.

### Autologous EBV-CTLs:

Whereas donor-derived EBV-CTL has been shown to be efficacious in the post HSCT setting, SOT recipients who develop EBV-LPD have different challenges, such as lack of donor availability and continued immune suppression. To overcome these challenges, several groups have used autologous EBV-CTLs in SOT recipients with EBV reactivation. While the *in vivo* persistence of CTLs was less than seen in donor-derived EBV-CTLs, infusions of autologous CTLs have been shown to be safe and no organ rejection occurred in patients receiving the CTLs^33^–^36^. Clinical responses have been seen but the response rate is lower than in PTLD after HSCT, likely reflecting decreased activity of CTLs in the presence of continuing immunosupression^35^,^36^.

### Third-party EBV-CTLs:

The primary downside to EBV-CTL generation for a specific patient is that it is expensive and time-consuming, taking up to three to four months to generate a suitable CTL line. Therefore, investigators have now generated banks of allogeneic virus-specific CTL lines from normal donors, so that most closely matched CTLs are available for patients in need of virus-specific immune reconstitution^37^. One concern with this “off the shelf” approach is that the recipient may generate an immune response to a non-shared HLA antigen. In a Phase II study evaluating this approach these third-party CTLs were used to treat EBV-LPD after HSCT or SOT with encouraging results: 64% response at 5 weeks and 52% at 6 months, with better responses noted in patients most closely HLA matched to the CTLs^37^,^38^. However for certain tumor types complete responses occurred in the absence of detectable specific CTL/tumor recognition perhaps because the population could not be detected or possibly because CTLs may have stimulated nonspecific inflammatory responses in vivo^38^. This strategy continues to be evaluated in clinical trials.

### Rapid Selection of EBV CTLs:

There are two alternative strategies being evaluated in early phase trials that can be used to rapidly reconstitute an EBV-specific immune response in the allogeneic HSCT setting. The first is to capture donor cells that secrete γ-IFN in response to antigenic stimulation. This approach can be used regardless of HLA type and captures both CD4 and CD8 T cells, but requires the donor to be available for pheresis. Another rapid selection strategy is to use magnetically-labeled peptide tetramers to select T cells specific for an EBV epitope. This approach has shown promise when used to reconstitute immunity to CMV^39^ but has the disadvantage of requiring knowledge of peptide epitopes suitable for each patient’s HLA type.

## Immunotherapy For Type Ii Latency Tumors:

Type II latency EBV-associated lymphomas occurring in individuals who do not have a known immunodeficiency include NK and T malignancies with cytotoxic phenotypes, and sporadic cases of B-NHL.^5^ Hodgkin’ disease is also associated with expression of EBV-derived antigens in malignant Reed-Sternberg (RS) cells in up to 50% of cases^4^,^40^. While HD can be very curable (with disease-free survival approaching 80–90%,^41^ survival is very poor for those who fail salvage chemotherapy or relapse multiple times. Thus, it is desirable to develop novel therapies to increase survival in patients with relapsed/refractory disease. EBV+ve NK and T malignancies respond poorly to standard chemotherapy and radiotherapy justifying exploration of strategies targeting EBV.

## Antibody Therapies for Type II Latency Lymphomas

### CD25 and CD30 Antibodies:

Monoclonal antibody therapy targeting the cell surface antigens, CD25 and CD30, present primarily on malignant RS cells could be a very attractive immunotherapy approach for HD. These monoclonal antibodies can be chemically linked to an active toxin such as *Pseudomonas* endotoxin A or deglycosylated ricin A^42^,^43^. Initial studies were limited by the immune response against murine antibodies and the toxin component but studies with humanized antibodies are now underway^44^.

## T-Cell Therapies for Type II Latency Lymphomas

### Unmanipulated Allogeneic T Cells:

As with type III latency EBV-LPD, DLI can be used for treatment of patients with type II latency HD or NHL following allogeneic HSCT^45^–^47^. One group administered DLI to 16 patients with residual disease or disease progression following transplant, with nine disease responses (including eight complete responses). However, high rates of GvHD were noted in the responders (six severe, acute GvHD and five chronic GvHD) 48. Another group reported a 44% response rate in nine patients with advanced HD who received DLI for persistent or progressive disease and all but one developed GvHD following DLI^49^. Thus, further evaluations of DLI approaches in these patients with difficult to treat disease is warranted however, developing strategies to maximize efficacy while minimizing toxicity is crucial.

### EBV-specific CTLs:

In type II latency EBV-HD and NHL, viral gene expression is limited to immunosubdominant proteins, including LMP1 and LMP2, which are weak targets for CTL activity, thereby allowing malignant cells to evade the immune system. Immunotherapy targeting these subdominant EBV antigens has been undertaken with some success, in both the autologous and allogeneic setting.

Our group initially evaluated the use of autologous polyclonal EBV-CTLs in 14 patients with relapsed EBV-HD, retrovirally marking CTL in seven patients. Five patients achieved complete remissions (two with detectable disease at time of CTL infusion), one achieved a partial response and five had stable disease^50^. Tetramer and functional analyses revealed that T cells reactive with LMP2 were present in the infused lines, expanded *in vivo* and could track to the sites of disease. The gene-marking studies proved that the infused cells could further expand by several logarithms with persistence up to 12 months^50^.

Since these studies used EBV-CTL which contained only low frequencies of T-cells specific for the tumor associated antigen LMP2, we then focused efforts on using genetically modified tumor antigen presenting cells that overexpress LMP2 as a strategy to increase the frequency of LMP2-specific T-cells in the product administered to patients. To accomplish this, we used dendritic cells that were engineered to express LMP2 using an adenovirus vector (Ad5f35LMP2A) for the primary stimulation, and then used LCLs modified with the same Ad5f35LMP2A vector for subsequent stimulations. Clinically, these LMP2-spcific CTL have been used in a dose-escalation study for 16 patients with high-risk EBV-HD and NHL^51^. Ten patients received CTLs as adjuvant therapy with nine remaining in complete remission for up to four years. Five of six with active, relapsed disease at time of infusion showed disease response (four complete) sustained for more than nine months. No toxicities have been observed after CTL infusion.^51^ To broaden this approach, we are now extending this strategy by using autologous T cells enriched for both LMP2 and LMP2, in a clinical trial is currently underway.

Because it is difficult to generate autologous CTL in sufficient quantity for heavily pre-treated patients, partially HLA-matched allogeneic CTL have been generated for a phase I study in patients with relapsed EBV-HD^52^. Five of six patients had a reduction in measurable disease up to 22 months. However, this approach was limited by the short-term persistence of the allogeneic T cells since donor-derived T cells could not be detected *in vivo*.

### Artificial T-cell Receptors:

Subpopulations of EBV-HD tumor cells may lack or lose expression of the weakly immunogenic antigens, such as LMP1 and LMP2, thus allowing tumor escape and treatment failure with CTLs. The genetic modification of human T cells to express tumor antigen-specific immune receptors offers a potential means of targeting other tumor associated antigens in addition to EBV. One approach is to use engineered T cell αβ-receptors which can be cloned from autologous CTL cultures or generated in HLA A2 transgenic mice but this is limited by HLA-type and generally confined to HLA-A2 donors. In addition inadvertent pairing between the native TCR and the transduced αβ chains may limit antitumor effects and cause off target side effects. A second approach is to incorporate chimeric antigen receptors (CAR) made of the antigen combining domains of antibody heavy and light chains, usually coupled to the intracellular components of the T cell receptor zeta chain to permit signal transduction after T-cell receptor engagement.

Most CAR-modified T cells have limited expansion, persistence and activity in vivo because they are inadequately co-stimulated. For CARs, it may be possible to overcome this limitation by the further incorporation of the endodomains of T cell co-stimulatory molecules such as CD28, OX40L or 4-1-BB. Alternatively, EBV specific CTL may be used as a CAR platform, since these cells retain long-term functionality in vivo and should receive all appropriate co-stimulation through their native receptors when they encounter viral antigens on normal antigen presenting cells, improving expansion and persistence and permitting subsequent killing of tumor cells through their chimeric receptor directed to a tumor associated antigen. A recent study confirmed that EBV CTLs may survive longer than T cells when grafted with a CAR perhaps due to the additional costimulation received through their native receptor^53^.

One trial with T cells transduced with a CAR specific for CD20 has been reported and several trials with CARs targeting CD19 are underway^54^,^55^. In Hodgkin’s Disease CD30, which is highly expressed on malignant RS cells is a target^56^ and in preclinical studies CD30 CAR+ EBV-CTLs retain their ability to kill EBV-positive lymphoma cells and have the ability to recognize and kill CD30+ HD tumor cells *in vitro* and *in vivo* in a severe combined immunodeficiency murine model.

## Immunotherapy For Burkitt Lymphoma – Type I Latency Tumors:

While many Burkitt lymphoma tumors are EBV positive, these are amongst the least immunogenic of the EBV-related tumors as they express a type I latency pattern (EBNA1 is on the only latent protein of the virus present and EBV gene expression is otherwise limited to the EBERs). EBNA1 is a challenging target for CTL as it possesses the unique glycine-alanine repeat (Gar) sequences that inhibit the endogenous presentation of CD8+ T-cell epitopes through the class I pathway by blocking proteasome-dependent degradation of EBNA1. However, since EBNA1 specific CD4+ T cells can be detected in healthy donors^57^, this antigen is a potential immunotherapeutic target. Several MHC class II restricted peptides from EBNA1 have been identified that recognized by CD4+ T cells and the potential use of these cells for adoptive immunotherapy is being explored^58^–^60^. Additionally, most Burkitt lymphoma tumor cells express CD20 on their surface, making them targets for rituximab therapy (monoclonal antibody directed against CD20).

## Conclusions:

Adoptive immuno-therapy, ranging from simple B-cell antibodies to complex and time-consuming CTL therapies, offers a potentially curative approach to many patients with EBV-related malignancies. Given that these treatments are usually reserved for relapsed or refractory patients, responses vary ranging from good complete responses to stable active disease. As researchers optimize the generation of these cells *ex vivo* allowing for enhanced *in vivo* persistence and expansion, we will hopefully begin to see more durable responses in this heavily pretreated population.
